# Genetically inducible and reversible zebrafish model of systemic inflammation

**DOI:** 10.1242/bio.058559

**Published:** 2022-03-09

**Authors:** Kevin A. Lanham, Megan L. Nedden, Virginia E. Wise, Michael R. Taylor

**Affiliations:** 1 Pharmaceutical Sciences Division, School of Pharmacy, University of Wisconsin, Madison, WI 53705, USA; 2 Currently at School of Medicine and Public Health, University of Wisconsin, Madison, WI 53705, USA; 3 Currently at PPD, Inc., Middleton, WI 53562, USA

**Keywords:** Inflammation, Zebrafish, Interleukin-1β, Tebufenozide, Neutrophil, Gal4-EcR/UAS

## Abstract

The inflammatory response is a vital defense mechanism against trauma and pathogen induced damage, but equally important is its appropriate resolution. In some instances of severe trauma or sustained infection, inappropriate and persistent activation of the immune response can occur, resulting in a dangerous systemic inflammatory response. Untreated, this systemic inflammatory response can lead to tissue damage, organ shutdown, and death. Replicating this condition in tractable model organisms can provide insight into the mechanisms involved in the induction, maintenance, and resolution of inflammation. To that end, we developed a non-invasive, inducible, and reversible model of systemic inflammation in zebrafish. Using the Gal4-EcR/UAS system activated by the ecdysone analog tebufenozide, we generated transgenic zebrafish that allow for chemically induced, ubiquitous secretion of the mature form of zebrafish interleukin-1β (Il-1β^mat^) in both larval and adult developmental stages. To ensure a robust immune response, we attached a strong signal peptide from the *Gaussia princeps* luciferase enzyme to promote active secretion of the cytokine. We observe a dose-dependent inflammatory response involving neutrophil expansion accompanied by tissue damage and reduced survival. Washout of tebufenozide permits inflammation resolution. We also establish the utility of this model for the identification of small molecule anti-inflammatory compounds by treatment with the immunosuppressant rapamycin. Taken together, these features make this model a valuable new tool that can aid in identifying potential new therapies while broadening our understanding of systemic inflammation, its impact on the immune system, and its resolution.

## Abstract (precis)

**Summary:** A new transgenic zebrafish model enables the regulated induction and resolution of systemic inflammation.

## INTRODUCTION

Inflammation is a complex physical and chemical response mounted by the body to respond to, and resolve, a perceived threat (Lord et al., 2014; Rock et al., 2010). These threats are sensed by interpreting signals released from injured cells, also known as damage-associated molecular patterns (DAMPS) (Rock et al., 2010), or by interpreting signals released from invasive microbes, known as pathogen-associated molecular patterns (PAMPS) (Medzhitov and Janeway, 1997). Based on the source of these signals, inflammation can be classified as either sterile or non-sterile in nature. In either case, recognition of these molecular patterns triggers the release of inflammatory cytokines including tumor necrosis factor alpha (TNFα), interleukin-6 (IL-6), and interleukin-1 beta (IL-1β) (D'Elia et al., 2013; Zeytun et al., 2010). These cytokines then act on other cell types, prompting the release of chemokines to attract and activate cellular effectors of the innate immune system (D'Elia et al., 2013; Sokol and Luster, 2015).

Neutrophils are one such effector. They are the first immune cell to arrive following an inflammatory event and are well equipped to deal with either sterile or microbial threats (de Oliveira et al., 2016; Nathan, 2006). As phagocytic cells, neutrophils can facilitate the removal of cellular debris at sterile wounds, or consume invading bacteria and viruses. They can also release granules containing lytic enzymes and emit bursts of reactive oxygen species (ROS), which damage and destroy pathogens. More recent studies have shown that neutrophils can also discharge a mesh of DNA and histone, known as neutrophil extracellular traps (NETs), which can entangle and encapsulate threatening microbes (Brinkmann et al., 2004). Given their potency, these cells are tightly regulated as their anti-microbial attacks can also damage the host (Bian et al., 2012; Weiss, 1989). Indeed, when an inflammatory insult cannot be so readily resolved, neutrophils may persist and, in severe cases, contribute to the inflammatory damage, resulting in a dangerous positive feedback loop of chronic, systemic inflammation with potentially fatal consequences (Bian et al., 2012; D'Elia et al., 2013; Pruitt et al., 1995). Understanding how this rampant systemic inflammatory response occurs and how it is resolved remains an area of intense interest, and animal models continue to play an important role in this regard (Seemann et al., 2017).

For this reason, we present here a zebrafish model of systemic inflammation designed to contribute to our understanding of this condition and facilitate the discovery of new treatment strategies. Zebrafish are a versatile vertebrate model animal that have contributed to our understanding of developmental processes (Chi et al., 2008; Herbert et al., 2009; Nicoli et al., 2010; Umans et al., 2017; Wienholds et al., 2005), and disease mechanisms (Langenau et al., 2003; Lopez-Rivera et al., 2017; Tobin et al., 2012; Walters et al., 2010). A variety of inflammation models have been developed using zebrafish and they encompass both DAMP and PAMP mediated signaling mechanisms. These include tailfin-resection (Renshaw et al., 2006; Yoo and Huttenlocher, 2011) and stab-wound (Cvejic et al., 2008; Mathias et al., 2006; Yoo and Huttenlocher, 2011) models to study inflammation caused by sterile injury, and infection with lipopolysaccharide (LPS) (Philip et al., 2017), bacteria (Deng et al., 2012; Hall et al., 2012; Le Guyader et al., 2008), fungi (Davis et al., 2016; Voelz et al., 2015), or viruses (Goody et al., 2017) to explore pathogen-mediated inflammatory processes. The ease of genetic manipulation in zebrafish has also given rise to several transgenic models that express inflammatory cytokines. Examples of the latter include heat-shock-inducible mature Il-1β (Yan et al., 2014), cell-specific expression of mature Il-1β (Delgadillo-Silva et al., 2019), and recently a doxycycline-inducible model driving expression of three inflammatory cytokines (Ibrahim et al., 2020).

Although these models are all useful, none of them fit our particular objective, which was to develop a model of systemic inflammation that is non-invasive, inducible and reversible. These criteria ruled out the physical manipulation inherent in wound or injection-based models of inflammation. A transgenic approach seemed most appropriate and although the previously published heat shock model met our requirements, we were concerned about the potentially confounding effects of heat shock (Lam et al., 2013), and the inability to drive tissue-specific expression if desired. Another approach to gene induction is the well-established TetON system, which our lab has used in the past (Ju et al., 2015); however, consistent with published reports (Huang et al., 2005), we have found it to be poorly reversible.

As an alternative, we used the Gal4-EcR/UAS system (Esengil et al., 2007), which provides several advantages and overcomes many of the limitations found in current zebrafish inflammation models. Using a ubiquitous promoter sequence (Mosimann et al., 2011) to drive expression of Gal4-EcR and the UAS promoter to drive active secretion of mature Il-1β fused to the *Gaussia princeps* luciferase signal peptide (Stern et al., 2007), we generated double transgenic zebrafish *ubb:IVS2GVEcR, UAS:GSP-il1b^mat^*. We show that our transgenic inflammation model promotes tebufenozide-induced, dose-dependent systemic inflammation that causes neutrophil expansion, tissue damage and increased mortality. We also show that these inflammatory effects are reversible simply by removing the inducing compound, tebufenozide. Finally, we demonstrate that the immunosuppressant drug rapamycin reduced neutrophil burden and promoted survival, highlighting the potential of this model to identify new drugs to treat systemic inflammation.

## RESULTS

### Design and generation of a transgenic, tebufenozide-inducible inflammation model

Since Il-1β has been validated as a strong inducer of inflammation in humans (Pruitt et al., 1995), as well as in other zebrafish model systems (Delgadillo-Silva et al., 2019; Ogryzko et al., 2014; Yan et al., 2014), we incorporated it in our model with some modification. Interleukin-1β is transcribed in a pro-form that requires proteolytic processing prior to secretion (Lopez-Castejon and Brough, 2011; Vojtech et al., 2012). Mature IL-1β lacks a signal peptide and its secretion mechanism is poorly understood, although it is generally thought to occur through microvesicles, exosomes, or passively following the death of inflamed cells (Lopez-Castejon and Brough, 2011; Vojtech et al., 2012). Vojtech et al., 2012 showed that the zebrafish Caspase 1 orthologs, Caspase A, and Caspase B, are involved in the processing and secretion of pro-Il-1β into its mature form. Previous models have used the putative mature form of zebrafish Il-1β, as we have; however, since we are bypassing the normal inflammatory cascade responsible for *il1b* expression and release, we attached a signal peptide derived from *Gaussia princeps* luciferase (Stern et al., 2007) to promote active secretion of mature Il-1β (GSP-il1b^mat^).

**Fig. 1.**
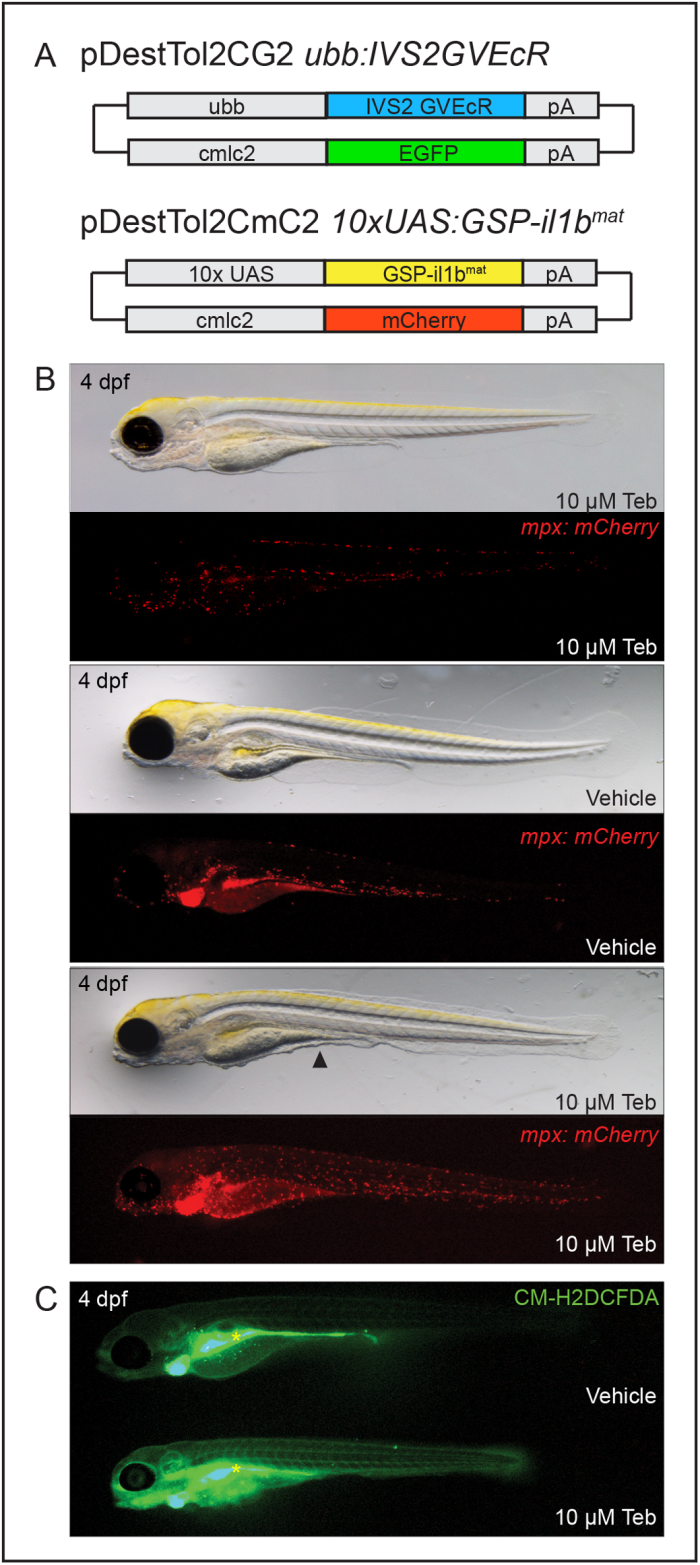
**Genetic induction of ubiquitous GSP-Il-1β^mat^ by the ecdysone mimic tebufenozide produces systemic inflammation.** (A) Schematic of DNA constructs injected to make the *Tg(ubb:IVS2GVEcR, cmlc2:EGFP)* and *Tg(UAS:GSP-il1b^mat^, cmlc2:mCherry)* lines. Driver lines and responder lines are distinguished by green *(cmlc2:EGFP)* or red *(cmlc2:mCherry)* hearts, respectively. (B) Transgenic larvae, expressing the driver and responder transgenes in the Casper *mpx:mCherry* background, were induced with vehicle (DMSO) or 10 µM Teb at 2 dpf. Fluorescent stereoscope images taken at 4 dpf show neutrophils spreading throughout Teb-induced larvae, while brightfield images reveal a sickly appearance and degradation of the median fin fold (black arrowhead). Casper *mpx:mCherry* larvae exposed to 10 µM Teb and vehicle exposed Casper *mpx:mCherry* larvae positive for both driver and responder transgenes appear normal; *n*=20 for each condition. (C) Larvae were exposed to vehicle or 10 µM Teb at 2 dpf and examined for the formation of ROS using the cell permeant indicator CM-H2DCFDA at 4 dpf. Larvae from each condition were imaged in the same field by fluorescence stereomicroscopy, revealing heightened ROS production in Teb-induced larvae; *n*=5 for each condition. The gut rapidly absorbs and forms fluorescent product in both larvae (yellow asterisk).

Since our objective was to make a model of systemic inflammation, we used the established zebrafish *ubiquitin b* (*ubb*) promoter (Mosimann et al., 2011) to achieve ubiquitous expression (Fig. 1A). This promoter then drives a modified transcription factor, which contains a fusion of the yeast Gal4 DNA binding domain, a truncated transactivation domain from the herpes simplex virus regulatory protein VP16, and a modified version of the ligand binding domain of the ecdysone receptor from the silk moth *Bombyx mori* (GV-EcR) (Esengil et al., 2007). The second intron of the rabbit β-globin gene (IVS2) was included to further enhance gene expression (Buchman and Berg, 1988; Esengil et al., 2007).

The GV-EcR protein functions as a ligand activated transcription factor; it is latent until activated by the insect hormone ecdysone, or a chemical analog, such as tebufenozide. Upon activation, it traffics to the nucleus where it binds a 10X yeast UAS that is attached to the carp beta-actin promoter and drives expression of the interleukin-1β cassette, *GSP-il1b^mat^*. Transgenesis reporters were included on driver and responder constructs, *cmlc2:EGFP* and *cmlc2:mCherry*, respectively, to facilitate identification of positive lines (Fig. 1A). Since neutrophils respond robustly to inflammatory signals through increased motility and expansion (Hall et al., 2012; Yoo and Huttenlocher, 2011), the transgenic lines were generated in a Casper *mpx:mCherry* background to provide a rapid readout of inflammation. Several founder lines were established and F1 animals that produced offspring in Mendelian ratios were identified and maintained.

As an initial test of our system, embryos heterozygous for each transgene were induced with 10 µM of the ecdysone analog tebufenozide (Teb) at 2 days post-fertilization (dpf) and examined for an inflammatory response. By 4 dpf, induced larvae showed degradation of the fin folds, most notably the anterior medial fin fold, and a generally sickly and emaciated appearance relative to vehicle treated controls (Fig. 1B). This phenotype was accompanied by an extensive expansion in neutrophils throughout the larvae. In contrast, neutrophils in control larvae were largely restricted to the caudal hematopoietic tissue (CHT) and scattered along the ventral trunk and head region, indicating that the *GSP-il1b^mat^* construct has no appreciable leakiness. Similarly, exposure of Casper *mpx:mCherry* embryos to 10 µM Teb in the absence of driver and responder transgenes produced no evidence of inflammation or developmental abnormalities. The formation of reactive oxygen species (ROS) is another indicator of inflammation (Niethammer et al., 2009). Using the same induction regimen, we tested whether ROS was increased in induced animals relative to controls using CM-H2DCFDA, a fluorescent ROS indicator. As shown in Fig. 1C, we found a qualitative increase in ROS formation in Teb-induced larvae.

### Tebufenozide induces GSP-Il-1β^mat^-mediated inflammation in a dose-dependent manner

**Fig. 2.**
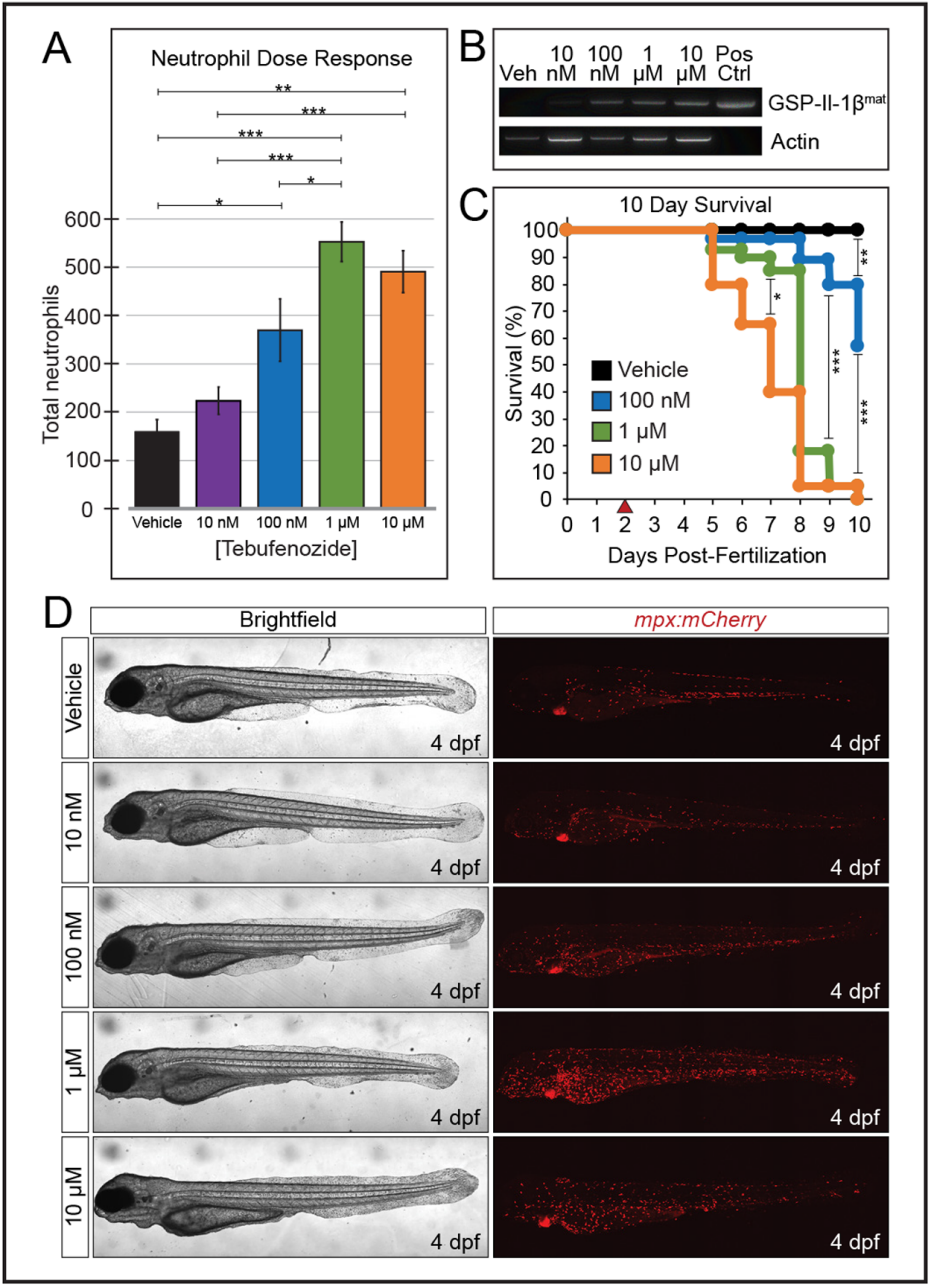
**GSP-Il-1β^mat^ driven systemic inflammation is dose dependent.** (A) Dose-dependent effects of Teb on neutrophil numbers following 2 days of exposure to either vehicle (DMSO) or increasing concentrations of Teb (*n*=6 for each concentration, one way ANOVA with Tukey HSD). (B) RT-PCR of *GSP-Il-1β^mat^* and *actb1* (Actin) at 4 dpf following exposure of embryos to vehicle or the indicated concentration of Teb at 2 dpf. Amplification of linearized plasmid DNA (25 pg) was included as a positive control. (C) Embryos were exposed to vehicle or indicated concentrations of Teb at 2 dpf (red arrowhead) and counted daily for 10 days to obtain Kaplan–Meier survival curves for each cohort, (*n*=40 for each concentration). (D) Representative images of larvae from (A) at indicated concentrations of tebufenozide. Brightfield images are minimum intensity projections, and mCherry images are maximum intensity projections of confocal stacks acquired for neutrophil counts. For all figures data are shown as the mean±s.e.m. (*P<0.05, **P<0.01, ***P<0.001).

To determine the sensitivity of our model and whether we could produce a graded inflammatory response, we tested a range of Teb concentrations (10 nM to 10 µM) and assessed inflammation by counting the total number of neutrophils after 48 h of exposure. Total neutrophil numbers increased in a concentration-dependent fashion with 100 nM, 1 µM and 10 µM doses differing by 2–4-fold from control animals (Fig. 2A). Consistent with the phenotypic dose response, the induction of the *GSP-il1b^mat^* transgene increased with escalating Teb concentrations as assessed by RT-PCR (Fig. 2B). The 10 nM dose of Teb, although seeming to provoke a mild inflammatory response, ultimately produced no significant change in neutrophil numbers. Interestingly, induction with 10 µM Teb increased neutrophil numbers, but in a range between the 100 nM and 1 µM concentrations, and not significantly different than either one individually. Given the sickly appearance of larvae following 10 µM Teb induction during our initial testing, we decided to analyze survival over a 10-day period at 100 nM, 1 µM, and 10 µM Teb concentrations (Fig. 2C). Ten-day survival also followed a dose-dependent response; however, contrary to what the neutrophil data might suggest, 10 µM Teb resulted in significantly worse survival than the 1 µM and 100 nM concentrations. Similarly, the 1 µM concentration fared worse than the 100 nM, which in turn had a significantly worse survival than the control. It is worth noting that our induction protocol begins at 2 dpf because induction with 1 or 10 µM Teb prior to 2 dpf results in nearly 100% mortality by 4 dpf (K.A.L., unpublished observations, data not shown). Representative brightfield and fluorescent images of larvae used to determine total neutrophil counts are shown in Fig. 2D. Larvae induced with 10 and 100 nM Teb appeared grossly normal following 48 h of induction. In contrast, larvae induced with 1 and 10 µM Teb showed degradative loss of the anterior medial fin fold and the emaciated appearance noted earlier.

### Systemic inflammation resolves after Teb washout

**Fig. 3.**
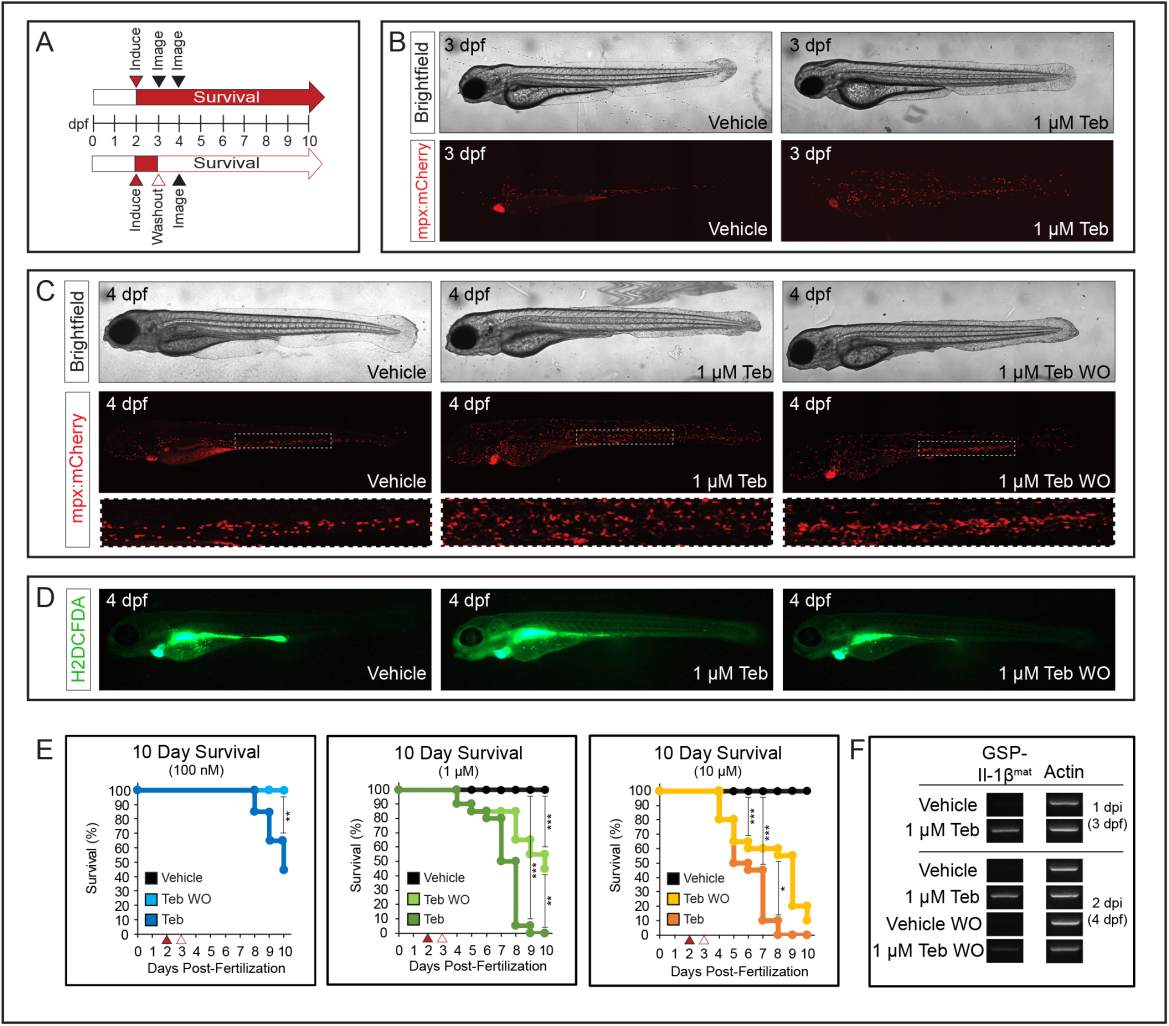
**Resolution of inflammation and increased survival following Teb washout.** (A) Schematic of experimental approach. Embryos were induced with vehicle (DMSO) or varying concentrations of Teb at 2 dpf (red arrowhead) and either maintained in, or washed to remove, Teb (open red arrowhead) at 3 dpf. Survival was determined daily over 10 days for Teb (red arrow) or washout (open red arrow). Representative images were taken at 3 and 4 dpf (black arrowheads). (B) Representative images of 3 dpf embryos, induced with vehicle or 1 µM Teb at 2 dpf, prior to washout. Tebufenozide induced embryos show increased expansion of neutrophils; *n*=20 for each condition. (C) Representative images of 4 dpf larvae showing vehicle treated, 48 h of 1 µM Teb exposure, and 24 h 1 µM Teb plus 24 h post-washout (WO). Dashed box shows expanded view of CHT region from each larva. Brightfield images are minimum intensity projections, and mCherry images are maximum intensity projections of confocal stacks; *n*=20 for each condition. (D) Increased formation of ROS shown using fluorescent indicator dye CM-H2DCFDA in 1 µM Teb-induced larvae relative to vehicle and washout larvae at 4 dpf; *n*=5 for each condition. (E) Ten-day Kaplan–Meier survival curves showing increased survival for larvae following Teb washout relative to persistent induction at the indicated concentrations of Teb. Embryos were exposed to vehicle, or indicated concentrations of Teb (red arrowhead) at 2 dpf, then either washed at 3 dpf (open red arrowhead), or maintained in the indicated concentration of Teb. Larvae were counted daily for 10 days to determine survival in each cohort; *n*=20 for each concentration. (F) RT-PCR showing induction of *GSP-Il-1β^mat^* relative to *actb1* expression (Actin) at 3 dpf and 4 dpf in embryos treated with vehicle or 1 µM Teb, with and without washout. (*P<0.05, **P<0.01, ***P<0.001).

**Fig. 4.**
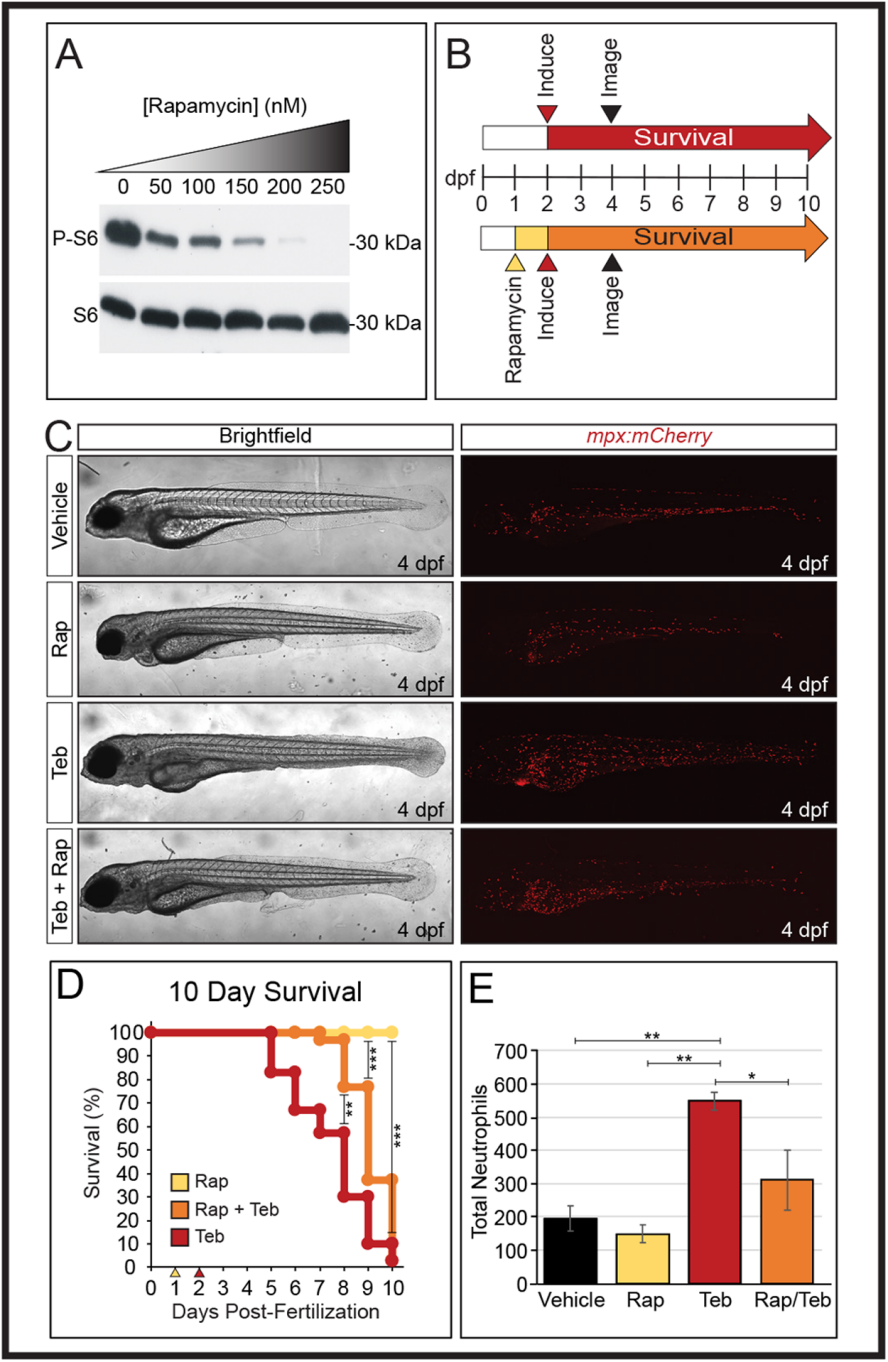
**Pre-treatment with the immunosuppressant rapamycin before genetic induction of ubiquitous GSP-Il-1β^mat^ alleviates systemic inflammation.** (A) Western blot showing dose-dependent response of ribosomal S6 phosphorylation to increasing rapamycin concentrations (0–250 nM) in zebrafish embryos. The level of total S6 was unaffected by rapamycin treatment. (B) Schematic of experimental approach. Embryos were treated with vehicle (DMSO) or 250 nM rapamycin (Rap) at 1 dpf (yellow arrowhead). At 2 dpf, embryos were induced with vehicle or 1 µM Teb (red arrowhead). Survival was determined daily over 10 days for vehicle, Teb (red arrow), and Teb plus rapamycin (orange arrow) cohorts. In a parallel experiment, larvae were imaged at 4 dpf (black arrowheads) and total neutrophil numbers determined. (C) Representative images of larvae at 4 dpf showing beneficial effects of rapamycin pre-treatment. Vehicle treated, 250 nM rapamycin (Rap) treated, 1 µM Teb, and 250 nM Rap+1 µM Teb-treated larvae are shown. Brightfield images are minimum intensity projections and mCherry images are maximum intensity projections of confocal stacks. (D) Ten-day survival curves showing increased survival for larvae pre-treated with 250 nM rapamycin (Rap) at 1 dpf (yellow arrowhead) followed by 1 µM Teb at 2 dpf (red arrowhead). Rapamycin treatment alone (yellow line) shows equivalent survival to vehicle controls (not shown); *n*=30 for each cohort. (E) Pre-treatment with 250 nM rapamycin (Rap) reduces total neutrophil number versus 1 µM Teb alone. Vehicle, Rap alone or Rap+Teb were not significantly different from each other; *n*=3 for each cohort (one way ANOVA with Tukey HSD). (*P<0.05, **P<0.01, ***P<0.001).

During a normal inflammatory response, neutrophils respond to and participate in resolving the inflammatory insult (Peiseler and Kubes, 2019). In our model, persistent Teb-dependent induction and secretion of Il-1β^mat^ prevents this resolution. Removal of Teb should terminate the production of Il-1β^mat^ and permit inflammation to resolve appropriately. To test this, we induced embryos at 2 dpf with varying concentrations of Teb (100 nM, 1 µM and 10 µM) to provoke an inflammatory response; then, after 24 h, we removed half of the embryos and washed them thoroughly before placing them in Teb-free water. Each population was then monitored daily for survival (Fig. 3A). As an initial assessment of inflammation resolution, we imaged embryos induced with 1 µM Teb at 3 dpf prior to washout to establish that inflammation had been induced (Fig. 3B), then again at 4 dpf to compare the appearance of vehicle treated, Teb induced, and Teb washout animals (Fig. 3C). Embryos at 3 dpf, following 24 h of induction with 1 µM Teb, showed widespread dispersal of neutrophils throughout the animal and increased neutrophils in circulation. These embryos also frequently displayed pericardial edema, which then progressed to the more emaciated appearance seen at 48 h post-induction. Embryos maintained in Teb showed the typical inflammatory response previously described for this model, including broad dispersal of neutrophils and degradation of the anterior medial fin fold. Interestingly, the latter effect occurred overnight as the fin fold was present at 3 dpf. In contrast, washout of Teb reduced the expansion and dispersal of neutrophils. The impact of washout on the fin fold was variable, ranging from mostly intact to degraded. We also noted that the CHT, which serves as a reservoir of neutrophils at this stage (Murayama et al., 2006), often showed an increased neutrophil density following Teb washout relative to control embryos, or to embryos continuously induced with Teb (Fig. 3C, dashed box). Given that Teb induction produced an increase in ROS along with the inflammatory response, we reasoned that upon washout we should see a reduction in ROS if inflammation is resolving. Consistent with this hypothesis, Teb-washout embryos showed reduced ROS induction compared to those maintained in 1 µM Teb (Fig. 4D). Further evidence of inflammation resolution is provided by the significant increase in 10-day survival following Teb washout at all concentrations examined (Fig. 4E). At the 100 nM concentration of Teb, post-washout survival was equivalent to un-induced control animals. At higher Teb concentrations, washout continued to produce a pronounced survival benefit relative to siblings maintained in Teb. As further confirmation of the effect of washout on Teb induction, we performed RT-PCR to analyze transgene induction at 3 dpf (1 day post-induction) and induction with or without Teb washout at 4 dpf (Fig. 4F). In agreement with the phenotypic response, we found strong induction of *GSP-il1b^mat^* at 3 dpf followed by continued or heightened induction at 4 dpf. Washout of Teb at 3 dpf resulted in reduced levels of transgene expression by the following day, consistent with the reduction in neutrophil number and ROS induction.

### Rapamycin mitigates the GSP-Il-1β^mat^-induced inflammatory response

Zebrafish inflammation models have previously been used to test anti-inflammatory compounds (Delgadillo-Silva et al., 2019; Robertson et al., 2014, 2016; Wang et al., 2014). As a proof of principle that our model can be similarly used to identify small molecule inhibitors of inflammation, we tested the prototypical immunosuppressant compound rapamycin to determine whether it could produce a survival benefit. Rapamycin acts on the well-conserved serine/threonine kinase mTOR, the mechanistic (or mammalian) target of rapamycin, which functions as a metabolic sensor and regulates cell proliferation, autophagy and protein translation in response to nutrient availability and stress (Inoki et al., 2012). As an indicator of mTOR activity, the phosphorylation state of one of its downstream targets, the ribosomal component S6, can be monitored (Chung et al., 1992). To examine the effectiveness of rapamycin in zebrafish we exposed larvae at 2 dpf to rapamycin (50–250 nM) overnight and analyzed S6 phosphorylation status by immunoblot. As shown in Fig. 4A, rapamycin inhibited phosphorylation of S6 in a dose-dependent manner in, whereas the level of un-phosphorylated S6 was unaffected by rapamycin treatment. Given this result, we pre-treated embryos with 250 nM of rapamycin at 1 dpf, 24 h prior to induction with 1 µM Teb (Fig. 4B). Images were taken at 4 dpf to assess total neutrophil numbers and the effects of treatment. In a parallel experiment, we determined 10-day survival. As can be seen in Fig. 4C, pre-treatment with rapamycin followed by 1 µM Teb mitigated many of the inflammatory effects produced by *GSP-il1b^mat^* induction, including fin fold degradation. Rapamycin exposure alone resulted in pericardial edema, but no other apparent defects, while 1 µM Teb produced the typical inflammatory response. Assessment of total neutrophil numbers following 48 h of 1 µM Teb induction, with and without rapamycin pre-treatment, revealed a substantial reduction in neutrophil counts with pre-treatment (Fig. 4D). Rapamycin alone did not significantly affect neutrophil numbers in the absence of an inflammatory trigger. Ten-day survival was similarly impacted by rapamycin pre-treatment resulting in a significant delay in mortality, but at day 8, a sudden increase in death in the rapamycin-treated cohort resulted in identical overall survival to the Teb-alone group (Fig. 4E). Larvae exposed to rapamycin alone had equivalent survival to vehicle-treated larvae.

### Non-invasive induction of inflammation in adult zebrafish

**Fig. 5.**
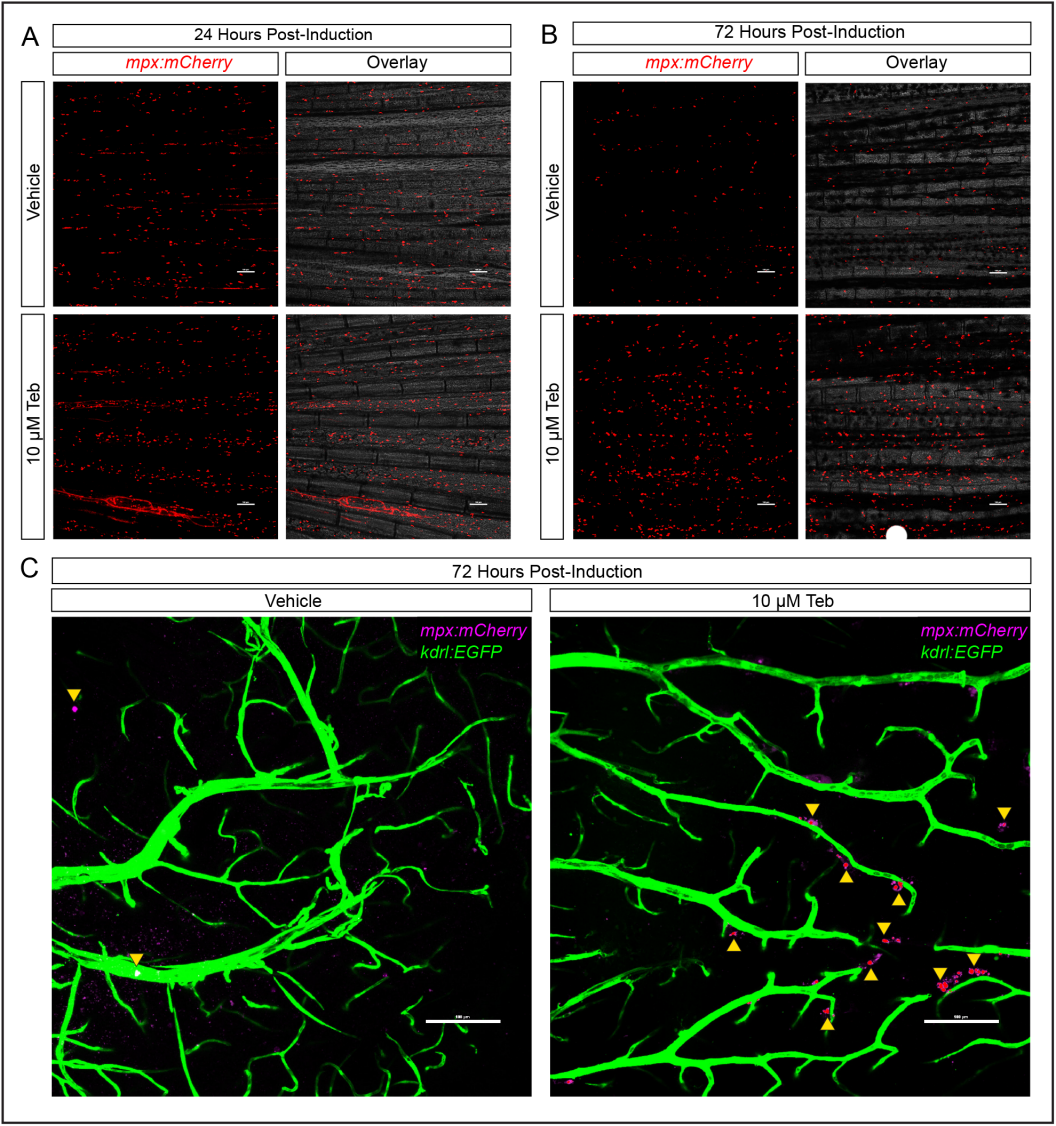
**Induction of GSP-Il-1β^mat^ in adult zebrafish triggers a robust neutrophil response.** (A) Rapid induction of inflammatory response in adults treated with 10 µM Teb versus vehicle (DMSO) demonstrated by non-invasive imaging of adult caudal fin 24 h post-induction. Maximum intensity projections of mCherry labeled neutrophils in vehicle treated or 10 µM Teb-induced adults are shown adjacent to image merged with minimum intensity projection of brightfield confocal stacks. (B) Continued inflammation at 72 h post-induction shown in adult fin by confocal microscopy. Maximum intensity projections of mCherry labeled neutrophils in vehicle treated or 10 µM Teb-induced adults are shown adjacent to image merged with minimum intensity projection of brightfield confocal stacks. (C) Inflammation on the surface of the optic tectum of adult zebrafish brain at 72 h post-induction with 10 µM tebufenozide relative to vehicle treated controls. Maximum intensity projections of confocal stacks show blood vessels labeled by *kdrl:EGFP* (green) and neutrophils labeled by *mpx:mCherry* (pseudo-colored magenta). Note the increased presence of perivascular neutrophils in Teb-induced animals (yellow arrowheads); *n*=6 for each condition.

The majority of immunological studies using zebrafish are performed on embryos and larvae, which is not surprising given their early transparency, rapid development, and ease of handling. Nevertheless, some zebrafish studies involving regeneration (Petrie et al., 2014; Poss et al., 2002), cancer (Dang et al., 2016; Kaufman et al., 2016), or the adaptive immune system (Langenau et al., 2004) have relied on the use of adult animals. To test whether we could induce an inflammatory response in adult zebrafish, we exposed 1-year-old male zebrafish, heterozygous for our driver and responder transgenes, as well as *mpx:mCherry* and *kdrl:EGFP*, to 10 µM Teb for 1 to 3 days by static, waterborne exposure. Male animals were used due to their increased availability relative to females in our colony and their overall consistency in size, which might otherwise influence Teb-mediated induction. Neutrophil numbers were monitored non-invasively by examining the tailfin for daily changes in neutrophil number using an epifluorescence stereomicroscope. Within 24 h, neutrophils were increased in the tailfin of Teb-exposed animals relative to controls (Fig. 5A) and were maintained at high levels until at least day 3 (Fig. 5B). On day 3, we removed the brains of vehicle and Teb-induced adults and imaged the optic tectum for changes in neutrophil abundance as evidence of inflammation. Perivascular neutrophils could be seen in increased numbers on the surface of the brain in Teb-induced animals consistent with an inflammatory response (Fig. 5C).

## DISCUSSION

We have developed a zebrafish model of inflammation that is non-invasive, inducible and reversible. We adopted the EcR-Gal4 system developed by (Esengil et al., 2007) to drive ubiquitous expression of a mature Il-1β protein fused to a signal peptide from *Gaussia princeps* luciferase to promote its active secretion. Since there are no endogenous ecdysone receptors in zebrafish, there is little likelihood of crosstalk with endogenous pathways that might complicate interpretation of the response. Consistent with this fact, we observed no apparent inflammatory or teratological effects of Teb exposure on embryos, even at the highest concentration used in this study. Another benefit of the ecdysone system is that the gene induction following administration of the ecdysone analog tebufenozide is easily reversible by washout, unlike other inducible systems such as the TetOn system (Huang et al., 2005).

We observed that induction of an inflammatory response was dose dependent, which allows for Teb concentrations to be titrated to achieve different levels of inflammation depending on the experimental goal. Low doses of Teb produced a mild inflammatory response resulting in moderate neutrophil expansion, while high doses caused severe systemic inflammation resulting in emaciated larvae with extensive neutrophil expansion and pronounced tissue damage. This is consistent with the analysis of transgene expression by RT-PCR showing a dose dependent induction of *GSP-il1b^mat^*. Total neutrophil numbers peaked at the 1 µM Teb concentration and were not further increased by induction with 10 µM Teb. It is reasonable that there would be a limit to the number of neutrophils that could be generated within a given time frame and that limit seems to have been reached. It could be contended that this plateauing effect is the result of saturation of Teb-dependent gene induction. Contrary to that point of view, the 10 µM concentration of Teb resulted in a significantly greater mortality than the 1 µM concentration, indicating that some unmeasured effector is still being altered by higher Teb concentrations.

Although neutrophil numbers plateau, it is still remarkable that the neutrophil population can expand so dramatically in such a short time. In zebrafish, neutrophil development can be followed from committed precursor to mature neutrophil by the expression of key transcription factors, granule components, and granule characteristics. Beginning with *cebp1* expression at 18 hpf, neutrophils progressively become positive for *mpx*, followed by *mpx*/*lyz*, before reaching maturity at ∼35 hpf with the development of granules that can be stained by Sudan Black (Jin et al., 2012; Le Guyader et al., 2008). Although the initial development of neutrophils is well established in zebrafish, we have found little to describe neutrophil kinetics under pathological conditions or during states of severe inflammation, particularly where total neutrophil numbers are assessed. There are two notable exceptions. The first exception is work by (Le Guyader et al., 2008) that demonstrated a 2-fold increase in neutrophils from 48 to 72 hpf in mutant zebrafish that lack a CHT, among other defects, which is a similar doubling rate to what we observed. Since they could no longer detect immature neutrophils that might contribute to this expansion and no new neutrophils could arise from the CHT, they concluded that mature, primitive neutrophils may retain some capacity to divide. The second exception is found in work by (Hall et al., 2012), in which *Salmonella enterica* was injected into the hindbrains of zebrafish embryos at ∼2 dpf. Strikingly, they noted a nearly total depletion of neutrophils at 1 day post-infection, but by 2 days post-infection counted nearly 800 *lyz* positive neutrophils, which were largely confined to the aorta-gonad-mesonephros and CHT. In contrast to (Le Guyader et al., 2008), where the primitive neutrophil population exists in the absence of a definitive one, the embryos infected by *Salmonella* appeared to lose their primitive population while maintaining their definitive one. In both cases, the neutrophil pool is capable of rapid expansion and replenishment, but each through different mechanisms. The larvae in our model retain their CHT, so it would be expected that neutrophils would be recruited from there; however, we also do not observe neutrophil depletion, so some contribution from the primitive population is also likely. The degree to which the primitive population contributes to the definitive population in the neutrophil expansion that we observe is yet to be determined.

Having established the non-invasive induction of inflammation, we wanted to demonstrate that our model was also reversible. Induction with 1 µM Teb at 2 dpf resulted in mild to moderate pericardial edema, accompanied by expansion of neutrophils across the embryo by 3 dpf, demonstrating that inflammation was present prior to Teb washout. Post-washout neutrophil expansion at 4 dpf was diminished relative to unwashed embryos, indicating that Teb mediated induction of *GSP-il1b^mat^* can be terminated and the inflammatory response resolved. The latter conclusion is further supported by the reduction in ROS and increased 10-day survival of these larvae. Though not quantified, the ROS reporter does not appear to reach baseline levels by 4 dpf and damage can be seen to the median fin fold in many larvae that have undergone washout, suggesting that inflammation persisted overnight and continued at a low level into the following day. In (Esengil et al., 2007), they showed that GV-EcR translocated out of the nucleus within 2 h of agonist removal, indicating a rapid deactivation of gene induction following washout. Notably, this effect was observed in cell culture and a live animal would likely take longer to clear the inducing agent. Consistent with this, RT-PCR revealed continued, but qualitatively less, expression of the *GSP-il1b^mat^* transgene 24 h following Teb washout relative to larvae without washout. Complete reversal of induction is also dependent on the half-life of the protein being expressed. Studies in rat indicate that IL-1β has a short serum half-life (Kudo et al., 1990), which would favor rapid resolution of the inflammatory response. In practice, the inflammatory process initiated by the secreted Il-1β^mat^ in our model, and the damage that it caused, took longer to resolve than anticipated. Indeed, although the 10-day survival of all cohorts undergoing washout was significantly increased relative to the unwashed cohorts, there was still significant mortality in all of the washout animals, except for the 100 nM group, relative to controls. Therefore, we conclude that even a brief period of systemic inflammation at this developmental stage can have long lasting and potentially fatal consequences.

Following Teb washout, we consistently observed an increased density of neutrophils in the CHT. Neutrophil reverse migration, as part of inflammation resolution, was first visualized *in vivo* in zebrafish (Mathias et al., 2006) and later observed by others both *in vitro* in humans (Hamza et al., 2014) and *in vivo* in mice (Woodfin et al., 2011). Recently, a mouse sterile injury model examined inflammation resolution and found neutrophils migrating from a wound site in the kidney to the lungs, where they upregulated CXCR4, before transiting to the bone marrow (Wang et al., 2017). In WHIM syndrome, constitutive CXCR4 activation causes neutrophils to be retained in the bone marrow (Kawai et al., 2007, 2005), while in a zebrafish model of WHIM, neutrophils are retained in the CHT (Walters et al., 2010). This homology suggests that the CHT could also serve a similar role to the bone marrow during inflammation resolution in the zebrafish; in which case, the increased density of neutrophils that we observed may be due to reverse migration and homing to the CHT. However, it should be noted that inhibiting Cxcr4, rather than activating it, has been shown to reduce neutrophil retention at sites of inflammation and promote reverse migration in zebrafish (Isles et al., 2019), indicating that Cxcr4 signaling may be downregulated during inflammation resolution. In mice, CXCR4 becomes upregulated in the lungs resulting in neutrophil migration to the bone marrow (Wang et al., 2017). No equivalent site or mechanism to trigger Cxcr4 activation and neutrophil migration to the CHT has been identified in zebrafish. Furthermore, neutrophils photo-converted at sterile wound sites in zebrafish, although undergoing reverse trans-endothelial migration, have not been observed to return to the CHT (Yoo and Huttenlocher, 2011). An alternate explanation for the increased density of neutrophils in the CHT is that emergency granulopoiesis (Hall et al., 2012) is activated in response to the strong inflammatory stimulus induced in our model. Since Teb washout removed that stimulus, neutrophils are no longer being actively recruited out of the CHT in response, and so accumulate within the hematopoietic tissue. Which mechanism is responsible for the increased density of neutrophils in the CHT following systemic inflammation resolution in this model, and the ultimate fate of these neutrophils, remains to be determined.

Zebrafish are demonstrably useful for small molecule drug screens in a variety of biomedically relevant conditions (White et al., 2011; Yu et al., 2008), including inflammation (Robertson et al., 2014, 2016; Wang et al., 2014). To establish the utility of our model in this capacity, we tested whether rapamycin, an inhibitor of mTOR and a prototypical immunosuppressant, could block the inflammatory response that we see following Teb induction. We first identified a waterborne concentration of rapamycin that could block phosphorylation of the S6 ribosomal subunit, a downstream target of mTOR activation, in zebrafish embryos. This provided confidence that the concentration we were using was functional and maximal. Exposure to rapamycin produced a slight developmental delay, and mild pericardial edema, consistent with previous reports of rapamycin exposure in zebrafish (Sucularli et al., 2016). Nevertheless, neutrophils developed in numbers equivalent to untreated control animals and 10-day survival was unaffected by rapamycin exposure. Rapamycin acts on neutrophils at multiple levels by inhibiting their proliferation, differentiation, and migration (Geest et al., 2009; Gomez-Cambronero, 2003; Rožman et al., 2015). Consistent with these effects, we observed a significant reduction in neutrophil numbers in larvae pre-treated with rapamycin prior to Teb induction. Rapamycin also significantly extended 10-day survival; however, at 8 dpf, a precipitous drop in survival occurred resulting in equivalent overall survival in both the Teb-induced and rapamycin/Teb cohorts. We did not refresh the rapamycin during the course of the experiment, and it is possible that the rapamycin became unstable or was metabolized by the larvae, resulting in a sudden release of inhibition at 8 dpf. Notably, tebufenozide is highly stable in solution (Sundaram, 1994), and would be continuously inducing secreted Il-1β^mat^ throughout the experimental time course. This could short circuit many of the usual feedback mechanisms that might alleviate the inflammatory state. The persistent induction of inflammation by Teb is a challenging obstacle to overcome. Use of this model in a drug screen may benefit from titration of Teb levels, or washout following induction to identify compounds that are anti-inflammatory but lack the extensive immunosuppressive properties of rapamycin.

While we focused our analyses on neutrophil responses for this study, there exist multiple transgenic lines that label other immune cell types including macrophages/microglia (Ellett et al., 2011; Walton et al., 2015), T-cells (Langenau et al., 2004, 2003), and B-cells (Page et al., 2013). Since we demonstrated an inducible inflammatory response in adult zebrafish, the opportunity now exists to explore systemic inflammation and the activation of the adaptive immune system, which does not develop until the juvenile stage (∼4–6 weeks) (Lam et al., 2004). Future work may also examine inflammatory responses using tissue-specific driver lines, in addition to responder lines employing different pro-inflammatory cytokines such as TNFα or IL-6. In addition, several questions were raised within this work with regards to the kinetics of neutrophil expansion and the resolution of severe systemic inflammation, which may be clarified with further experimentation in this model.

## MATERIALS AND METHODS

### Zebrafish husbandry, strains, and transgenic lines

All experiments were performed in accordance with the University of Wisconsin-Madison Institutional Animal Care and Use Committee. Zebrafish (*Danio rerio*) lines were maintained using guidelines set forth in The Zebrafish Book (Westerfield, 1993). Embryos and larvae were maintained at 28.5°C in egg water (0.03% Instant Ocean reconstituted in reverse osmosis water). The *Tg(mpx:mCherry)^uwm7^* line (kind gift from Dr. Anna Huttenlocher, UW-Madison) was bred into the *roy^a9/a9^, mitfa^w2/w2^* (Casper) background to generate the Casper *Tg(mpx:mCherry)* line. Homozygous *Tg(kdrl:EGFP^y1^)* (Cross et al., 2003) animals were bred into Casper lines heterozygous for the *ubb:IVS2GVEcR, UAS:GSP-il1b^mat^,* and *mpx:mCherry* transgenes and screened to obtain animals heterozygous for each transgene, which were used for adult (1 year old) Teb induction experiments. All other experiments used Casper animals heterozygous for the driver and responder transgenes *ubb:IVS2GVEcR, UAS:GSP-il1b^mat^* and heterozygous or homozygous for *mpx:mCherry*.

### Creation of Tg(ubb:IVS2GVEcR; cmlc2:EGFP) and Tg(UAS:GSP-il1b^mat^; cmlc2:mCherry) lines

Plasmids were constructed using a combination of PCR, Gateway cloning (Invitrogen) and components of the Tol2kit (Kwan et al., 2007). For construction of the pME:GSP-il1β^mat^ vector, total RNA was extracted from 5 dpf zebrafish larvae using Trizol (Thermo-Fisher) and cDNA was synthesized by RT-PCR using the Superscript III First-Strand Synthesis System (Invitrogen). This cDNA was used as template to attach the *Gaussia princeps* luciferase signal peptide to the presumptive mature Il-1β coding sequence (Vojtech et al., 2012) using three rounds of PCR with the following primer pairs: Round 1: *Gaussia* SP/il1b Forward (F): 5′-GTATTGCAGTCGCCGAAGCATCAGTGCCGTCTTACAATAAAACCAAAAACG-3′ plus AttB2R il1b Reverse (R): 5′-GGGGACCACTTTGTACAAGAAAGCTGGGTGCTAGATGCGCACTTTATCCTG-3′; Round 2: Gaussia SP F: 5′-ATGGGAGTTAAAGTGCTCTTTGCCCTGATATGTATTGCAGTCGCCGAAGCA-3′ plus AttB2R il1b R; Round 3: AttB1 Gaussia SP F: 5′-GGGGACAAGTTTGTACAAAAAAGCAGGCTGCCACCATGGGAGTTAAAGTGCTCTTTG-3′ plus AttB2R il1b R. The final product was recombined with pDONR221 (Invitrogen) using BP Clonase II (Invitrogen) to make pME-GSP-il1b^mat^. To construct the pME:IVS2GVEcR vector IVS2GVEcR was PCR amplified from myl7:IVS2GVEcR,UAS:GFP (kind gift from Dr. James Chen, Stanford University) using the following primer pair: AttB1 IVS2GVEcR F: 5′-GGGGACAAGTTT­GTACAAAAAAGCAGGCTCCGACCGATCCTGAGAACTTCAGG-3′ and AttB2r IVS2GVEcR R: 5′-GGG­GACCACTTTGTACAAGAAAGCTGGGTCCTATAGCACCACCGGGTTGGTG-3′ and recombined with pDONR221 as above. To make the pDestTol2CG2 ubb:IVS2GVEcR construct, p5E:ubb (generated as described in Mosimann et al., 2011), pME:IVS2GVEcR, p3E:pA and pDestTol2CG2 were recombined using LR Clonase II Plus (Invitrogen). To make the pDestTol2CmC2 UAS:GSP-il1b^mat^ construct, p5E:10X UAS, pME:GSP-il1b^mat^, p3E:pA, and pDestTol2CmC2 were recombined as above.

To generate the *Tg(ubb:IVS2GVEcR, cmlc2:EGFP)* and *Tg(UAS:GSP-il1b^mat^, cmlc2:mCherry)* transgenic lines, the final destination constructs were co-injected into Casper *Tg(mpx:mCherry)* single-cell embryos either individually or in combination (50–100 pg total plasmid DNA) together with 20 pg of *in vitro* transcribed *Tol2* transposase mRNA in a final volume of 1–2 nanoliters. Embryos with strong transient expression of the transgenes were raised to adulthood and screened for germline transmission. No change in the effectiveness of the driver and responder lines was observed out to the F4 generation.

### Microscopy

Zebrafish larvae were anesthetized in 0.02% Tricaine and immobilized in 1.2% low melting point agarose (Invitrogen) in a 35 mm glass-bottom dish, number 1.5 (MatTek). For adult tissues, zebrafish were euthanized in 0.02% Tricaine prior to dissection and imaging. Confocal microscopy was performed using a Nikon Eclipse Ti microscope equipped with a Nikon A1R. All confocal images are 2D projections of 3D confocal z stacks rendered using FIJI software (NIH) with either the maximum intensity projection algorithm (fluorescent images) or minimum intensity projection algorithm (brightfield images). For stereomicroscopy, larvae were anesthetized in 0.02% Tricaine and immobilized in egg water with 4% methyl cellulose (Sigma) in a 35×10 mm petri dish (Falcon). A Nikon SMZ18 epifluorescence stereomicroscope was used to capture images in brightfield or fluorescence using a Nikon DS-Fi2 color camera and processed using Nikon NIS-Elements software.

### Tebufenozide (Teb) induction, washout, and survival

Teb (Sigma) was made as a 10 mM stock solution in DMSO and stored at −20°C. Serial dilutions of the 10 mM stock were performed to create 1 mM, 300 µM, 100 µM and 10 µM stock solutions. Working concentrations (100 nM to 10 µM) were made by 1000-fold dilutions of master stocks into fresh egg water. Embryos in the indicated genetic backgrounds were induced with Teb at 2 dpf by static, waterborne exposure with the indicated concentrations and either washed out at 3 dpf or maintained in Teb for up to 10 days and each day survivors were tallied. For washout experiments, half of the Teb-induced embryos were transferred at 24 h post-induction to an empty 100×15 mm petri dish (Falcon) and residual liquid removed by micropipette. Embryos were then washed with 4×20 ml volumes of fresh egg water then transferred to a new 100×15 mm petri dish containing 20 ml of egg water. For survival curves, data was plotted using Excel (Microsoft) and Kaplan–Meier survival statistics calculated using the Real Statistics Resource Pack Excel add-in (www.real-statistics.com). For experiments involving adult animals, 1-year-old male Casper zebrafish [*Tg(ubb:IVS2GVEcR, cmlc2:EGFP)*, *Tg(UAS:GSP-il1bmat, cmlc2:mCherry)*, *Tg(mpx:mCherry)*, *Tg(kdrl:GFP)*] were placed in individual tanks containing 500 mLs of static water and then induced with vehicle (DMSO) or 10 µM Teb for 1 to 3 days.

### Reactive oxygen species (ROS) Assay

Twenty embryos per condition were Teb-induced at 2 dpf and either transferred to fresh egg water at 3 dpf for washout experiments or maintained in Teb until 4 dpf. Embryos were randomly selected from each plate and placed in egg water containing 10 µM CM-H2DCFDA (Invitrogen) and 0.02% Tricaine for 30 min in the dark. Animals were mounted in egg water with 4% methyl cellulose and imaged by epifluorescence stereomicroscopy.

### Rapamycin treatment

Rapamycin (Sigma) was prepared in DMSO (Sigma) at a stock concentration of 10 mM and stored in aliquots at −20°C. For inflammation and survival experiments, the rapamycin stock solution was serially diluted to 250 µM in DMSO, which was further diluted 1000-fold in egg water to 250 nM for embryo exposures at 24 hpf. Rapamycin was used individually or in combination with Teb followed by survival curves (described above) and counting the number of neutrophils.

### Western blot analysis

For western blot analysis, working concentrations of rapamycin (50 nM to 250 nM) were made fresh in egg water. Larvae at 2 dpf were treated with rapamycin for 24 h. Larvae were collected at 3 dpf (30 larvae/Rapamycin concentration) and homogenized in PBS with a protease inhibitor cocktail (Roche) and 1 mM EDTA on ice using a disposable homogenizer (Kontes). SDS sample buffer was added and the samples were boiled for 5 min. The equivalent of one larva per well were subjected to 12% SDS-PAGE. Following transfer to nitrocellulose, the blots were blocked in 5% skim milk, incubated with primary antibodies S6 (1:500; Cell Signaling #2217) and PS6 (1:2000; Cell Signaling #2211), followed by secondary antibodies conjugated to horseradish peroxidase (1:2000; Amersham), and visualized by Enhanced ChemiLµMinescence Plus (Amersham, NA934).

### RNA extraction, cDNA synthesis, and RT-PCR

Embryos were collected in triplicate at the indicated times and conditions, *n*=20 per replicate. Replicates were anesthetized in 0.04% Tricaine, washed with clean egg water in a microfuge tube and RNA extraction using TRIzol (Invitrogen) and chloroform extraction followed by ethanol precipitation. One microgram of total RNA was DNase treated using ezDNase (Invitrogen), followed by synthesis of cDNA using the Superscript IV First-Strand Synthesis System (Invitrogen) with Oligo(dT)_20_ primers according to the manufacturer's protocol. Each aliquot of cDNA was serially diluted 100-fold for use in RT-PCR. *GSP-il1b^mat^* was amplified using the AttB1 Gaussia SP F/AttB2R il1b R primer pair listed above. The expression of *actb1* was determined using actb1 F: CCCTCCATTGTTGGACGAC and actb1 R: CCGATCCAGACGGAGTATTTG. The pME:GSP-il1b^mat^ plasmid, linearized at BbsI, was used as a positive control. PCR was performed using GoTaq Hot Start Polymerase (Promega, M5005), with one microliter of diluted cDNA, or 25 picograms of linearized plasmid, per 20 µl reaction. Cycling conditions were: initial denaturation (94°C for 3 min), followed by 35 cycles of denaturation (94°C for 20 s.) annealing (55°C for 20 s.) and extension (72°C for 1 min). This was followed by a final extension (72°C for 5 min) then termination at 4°C. PCR reactions were performed at least three times, representative results are shown.

### Neutrophil quantification

Embryos were induced at 2 dpf with the indicated concentrations of Teb and imaged at 4 dpf or pre-treated with 250 nM Rapamycin and imaged at 4 dpf. Maximum intensity projections of z-stacks were constructed using FIJI software (NIH) then converted to 8-bit images. Under *Image>Adjust>Threshold* the upper threshold slider was placed at 75. Next, images were processed to account for neutrophil overlap using *Process>Binary>Watershed* to split adjacent cells. Cells were then counted under *Analyze>Analyze Particles* with size set to 20–200 and circularity to 0–100. Data was plotted with Excel (Microsoft) and analyzed by one-way ANOVA with Tukey HSD *post-hoc* test using the Real Statistics Resource Pack Excel add-in (www.real-statistics.com).
